# Epidemiologic characteristics of invasive group B streptococcal infections caused by rare serotypes among adults in the United States, 2007–2023

**DOI:** 10.1371/journal.pgph.0006205

**Published:** 2026-04-21

**Authors:** Sydney Haldeman, Namrata Prasad, Benjamin J. Metcalf, Allison Taffet, Stephanie J. Schrag, Adam J. Ratner

**Affiliations:** 1 Department of Pediatrics, New York University Grossman School of Medicine, New York, New York, United States of America; 2 Division of Bacterial Diseases, Centers for Disease Control and Prevention, Atlanta, Georgia, United States of America; 3 Department of Microbiology, New York University Grossman School of Medicine, New York, New York, United States of America; PLOS: Public Library of Science, UNITED STATES OF AMERICA

## Abstract

Using CDC’s Active Bacterial Core surveillance data, we observed increasing incidence of rare group B *Streptococcus* serotypes VI and VIII, from 0.007 and 0 cases per 100,000 in 2007 to 0.14 and 0.13 in 2023, respectively. These serotypes disproportionally impacted American Indian/Alaska Native, Asian, and Native Hawaiian/Pacific Islander populations.

## Introduction

Group B *Streptococcus* (GBS) is a common colonizer of the gastrointestinal and urogenital tracts and a leading cause of neonatal sepsis and meningitis. [1] It has also been recognized as an important cause of invasive disease among adults aged 65 years and older and those with underlying conditions such as diabetes mellitus and obesity. [2,3] In the United States, invasive GBS infections are monitored by the Centers for Disease Control and Prevention’s (CDC) Active Bacterial Core surveillance (ABCs) program, an active, laboratory- and population-based system across 10 sites. [4]

Maternal vaccines to reduce the burden of neonatal GBS disease are in development. A hexavalent anti-capsular polysaccharide conjugate GBS vaccine (GBS6) is currently in development and targets 6 of the 10 known serotypes (Ia, Ib, II, III, IV, and V), which account for approximately 98% of disease cases in neonates. [5,6] This vaccine may also help prevent GBS disease in adults, given that these vaccine-type serotypes (VT) have historically comprised approximately 99% of U.S. adult infections. [2] However, GBS serotype distributions vary worldwide and have changed over time. [1] Additionally, few surveillance systems have sufficient sample size to investigate disease cases caused by rare non-vaccine type GBS serotypes (NVT). We analyzed ABCs data on invasive GBS cases in the U.S. during 2007–2023 to describe NVT epidemiologic characteristics and compared them with VT serotypes.

## Methods

### Ethics statement

The Centers for Disease Control and Prevention (CDC) determined that this public health surveillance project was not human subjects research; therefore, CDC Institutional Review Board approval was not required.

### Surveillance and laboratory methods

ABCs conducts population-based surveillance for invasive group B *Streptococcus* (GBS) disease in defined catchment areas. Surveillance and laboratory methods for ABCs have been described previously. [2,4,7] In brief, a case is defined as isolation of GBS from a normally sterile site in a surveillance-area resident. Case finding is active and laboratory-based: ABCs personnel contact microbiology laboratories processing specimens for catchment-area residents and identify cases via laboratory calls and/or electronic line listings; routine audits are conducted to ensure complete case capture. For identified cases, ABCs requests submission of invasive isolates, which are shipped to CDC for laboratory characterization. The current analysis was limited to the seven ABCs sites (California [3 counties; 2015 onwards], Colorado [5 counties; 2011 onwards], Georgia [20 counties, Atlanta area], Maryland [entire state], Minnesota [entire state], New Mexico [entire state], and Oregon [3 counties, Portland area]) that forwarded GBS isolates from people of all ages to the CDC *Streptococcus* laboratory for serotyping. These areas represented an aggregate population of approximately 28 million according to the 2023 U.S. census. Serotypes were assigned by the CDC *Streptococcus* Laboratory using an in-house developed latex agglutination kit and the capillary precipitation test from 2007-2014 and predicted from whole-genome sequencing from 2015 onward. [8] Isolates for which a serotype was unable to be assigned through the latex agglutination method or whole-genome sequencing were categorized as non-typeable. A subset of serotype VI and VIII isolates underwent phylogenetic analysis (**S1 Text**). Demographic and clinical information, including clinical syndromes and underlying medical conditions, were abstracted from medical records using standardized case report forms. [7] Data were accessed on September 24, 2025. No personal identifying information about individual participants is available to authors. However, the data are not publicly available due to small case numbers in specific strata that could compromise the privacy of research participants.

### Statistical methods

Incidence rates (IRs) of invasive GBS disease were calculated as number of cases per 100,000 population using U.S. Census annual population estimates and were stratified by VT, NVT, and individual NVT serotype. Missing serotype data were accounted for in IR calculations using a single imputation process by assigning values to cases with missing data based on year, age group, and race-specific distribution of cases with known data. To assess changes in IRs between 2007 and 2023, incidence rate ratios (IRRs) were calculated with 95% CIs estimated using Poisson regression. Differences in the proportion of VT vs NVT by age, race (White, Black, American Indian/Alaska Native [AI/AN], Asian, Native Hawaiian/Pacific Islander [NH/PI], Multiracial), and clinical characteristics were assessed using Pearson χ2 or Fishers exact test. All statistical tests were considered statistically significant at a P value <0.05. Analyses were performed using the R statistical programming language version 4.0.4.

## Results

A total of 37,118 cases of invasive GBS disease were identified in the selected ABCs catchment areas during 2007–2023. Of cases, 5,310 (14.3%) lacked available isolate or serotype data. Among cases with serotype data, 2,944 cases were individuals aged <18 years and included 16 (0.5%) NVT cases. The small number of NVT cases aged <18 years limited analysis of NVT epidemiologic characteristics in this age group. As such, the remaining analysis and report focuses on 28,864 adult cases aged ≥18 years with serotype data. Of these cases, 532 (1.8%) were NVT cases, including 268 (50.4%) serotype VI, 133 (25.0%) serotype VIII, 65 (12.2%) non-typeable strains, 39 (7.3%) serotype IX, and 27 (5.1%) serotype VII.

**Fig 1:**
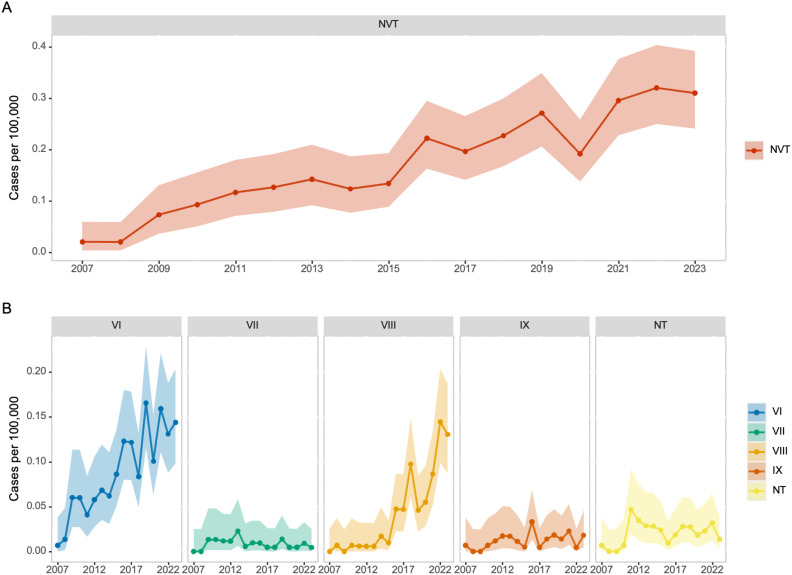
(A) Incidence rates of invasive GBS among U.S. adults by non-vaccine serotypes (NVT). (B) Incidence rates of invasive GBS among U.S adults by individual NVT serotypes. Shaded areas represent 95% confidence intervals.

NVT incidence rates among adults increased from 0.02 per 100,000 in 2007 to 0.31 per 100,000 in 2023 (IRR, 15.13 [95% CI, 5.64–61.81]) (Fig 1A). In contrast, the incidence of VTs increased from 7.85 per 100,000 in 2007 to 10.06 per 100,000 in 2023 (IRR, 1.28 [95% CI, 1.19–1.38]). Among NVTs, the incidence of serotype VI and serotype VIII increased the most, rising from 0.007 and 0 cases per 100,000 in 2007 to 0.14 and 0.13 per 100,000 in 2023, respectively (Fig 1B). While no single clone of serotypes VI or VIII predominated across sites, an exception was observed in the serotype VIII phylogeny, which contained a well-supported clade composed predominantly of isolates from the Maryland ABCs site. Of the 24 isolates within this clade, 22 originated from Maryland, with one isolate each from New Mexico and California (S1 and S2 Figs). While serotype VI incidence increased over the course of the study period, a rapid increase in the serotype VIII incidence was observed in recent years (Fig 1B).

AI/AN, Asian, and NH/PI individuals accounted for a greater proportion of NVT cases than VT cases (Table 1). Asian individuals accounted for 9.4% (50/532) of NVT cases while making up 2.0% (573/28,332) of VT cases (p < 0.001). This overrepresentation was observed specifically among serotype VI (10.8% [29/268]) and serotype VIII cases (8.3% [11/133]) compared to VT cases (p < 0.001 for both comparisons) (Table 2). Likewise, AI/AN and NH/PI individuals accounted for 8.6% (46/532) and 1.7% (9/532) of NVT cases, compared to 3.6% (1,021/28,332) and 0.3% (86/28,332) of VT cases (Table 1). Among serotype VI cases, AI/AN and NH/PI individuals accounted for a higher proportion of cases (14.9% [40/268] and 1.9% [5/268], respectively) compared to their VT counterparts (3.6% [1,021/28,332] and 0.3% [86/28,332], p < 0.001 and p < 0.002, respectively) (Table 2). No significant differences were observed between serotype VIII cases and VT cases among AI/AN and NH/PI individuals.

**Table 1:** Racial distribution among VT and NVT cases of invasive GBS infection in U.S. adults, 2007-2023.

Race	VT	NVT
	N = 28,332	N = 532
White	17997 (63.5%)	294 (55.3%)
Black	5423 (19.1%)	68 (12.8%)
AI/AN	1021 (3.6%)	46 (8.6%)
Asian	573 (2.0%)	50 (9.4%)
NH/PI	86 (0.3%)	9 (1.7%)
Multiracial	87 (0.3%)	4 (0.8%)
Missing	3145 (11.1%)	61 (11.5%)

*Note.* VT = Vaccine-Type. NVT = Non-vaccine Type. AI/AN = American Indian/Alaska Native. NH/PI = Native Hawaiian/Pacific Islander. All data displayed as n (%). N/A = Not Available.

**Table 2:** Characteristics of invasive GBS infection by serotypes VI, VIII, and VTs in U.S. adults, 2007-2023.

	VI	VIII	VT
	N = 268	N = 133	N = 28,332
**Age group (years)**			
18-39	20 (7.5%)	16 (12.0%)	2588 (9.1%)
40-64	106 (39.6%)	63 (47.4%)	12516 (44.2%)
65-79	89 (33.2%)	33 (24.8%)	8531 (30.1%)
80+	53 (19.8%)	21 (15.8%)	4697 (16.6%)
**Sex**			
Male	143 (53.4%)	75 (56.4%)	16348 (57.7%)
Female	124 (46.3%)	58 (43.6%)	11983 (42.3%)
Missing	1 (0.4%)	0 (0%)	1 (0.0%)
**Race**			
White	136 (50.7%) **	70 (52.6%)	17997 (63.5%)
Black	20 (7.5%) **	29 (21.8%)	5423 (19.1%)
AI/AN	40 (14.9%) **	4 (3.0%)	1021 (3.6%)
Asian	29 (10.8%) **	11 (8.3%) **	573 (2.0%)
NH/PI	5 (1.9%) **	2 (1.5%)	86 (0.3%)
Multiracial	3 (1.1%)	1 (0.8%)	87 (0.3%)
Missing	35 (13.1%)	16 (12.0%)	3145 (11.1%)
**Ethnicity**			
Hispanic	17 (6.3%)	15 (11.3%)	2346 (8.3%)
Non-Hispanic	187 (69.8%)	91 (68.4%)	18602 (65.7%)
Missing	64 (23.9%)	27 (20.3%)	7384 (26.1%)
**Outcome**			
Died	21 (7.8%)	10 (7.5%)	1844 (6.5%)
Survived	247 (92.2%)	123 (92.5%)	26400 (93.2%)
Missing	0 (0%)	0 (0%)	88 (0.3%)
**Systemic infection*^±^***			
Yes	127 (47.4%)	55 (41.4%)	11442 (40.4%)
No	141 (52.6%)	78 (58.6%)	16890 (59.6%)
**Septic shock*^±^***			
Yes	37 (13.8%)*	N/A	2438 (8.6%)
No	231 (86.2%)	N/A	25894 (91.4%)
**Diabetes**			
Yes	156 (58.2%)	75 (56.4%)	14850 (52.4%)
No	106 (39.6%)	54 (40.6%)	12514 (44.2%)
Missing	6 (2.2%)	4 (3.0%)	968 (3.4%)
**Obesity** *‡*			
Yes	123 (45.9%)	46 (34.6%)	13642 (48.2%)
No	140 (52.2%)	83 (62.4%)	13812 (48.8%)
Missing	5 (1.9%)	4 (3.0%)	878 (3.1%)

*Note. All comparisons shown are relative to VT group. VT = Vaccine-type serotypes. AI/AN = American Indian/Alaska Native. NH/PI = Native Hawaiian/Pacific Islander. All data n (%). All data n (%) unless otherwise noted. All p-values were calculated after excluding missing data. N/A = Not Available.*

*p < 0.01; **p < 0.001

*± Clinical syndrome data were obtained from information provided in medical records. Systemic infection was defined as a documentation of one or combination of the following during hospital admission: bacteremia without focus, septic shock, streptococcal toxic shock syndrome (STSS), or hemolytic uremic syndrome (HUS).*

*‡Obesity was determined through a combination of height and weight, body mass index (BMI), or medical record information.*

Serotype VI case-patients were slightly older compared to VT case-patients (mean age in years = 65.1 vs 62.6, p = 0.013). Septic shock syndrome was reported more frequently with serotype VI compared to VT cases (13.8% [37/268] vs 8.6% [2,438/28,332], p = 0.004). No significant difference was found between groups in terms of underlying conditions, case fatality, and other clinical syndromes when stratified by VT and NVT or by serotypes VI and VIII (**Table 2**).

## Discussion

While NVT-associated invasive GBS infections remained rare, their incidence has increased among U.S. adults during 2007–2023. We observed key differences in demographic characteristics between VT and NVT groups, with AI/AN, Asian, and NH/PI individuals disproportionally represented among NVT cases. Serotypes VI and VIII were the greatest contributors to the increase in NVT incidence across study years. We observed a sharp increase in the frequency in serotype VIII incidence beginning in 2016. Similar temporal increases have been reported in Canada, where serotype VIII accounted for a greater number of adult invasive GBS cases in 2014–2020 (N = 7) than in 2003–2013 (N = 3). [9,10] Notably, South Korea has reported serotype VIII emerging as the predominant serotype of adult invasive GBS disease (42.1% of cases) in recent years, compared to 11.1% from 2007-2009. [11] Based on evidence from phylogenetic and functional studies, serotype VIII GBS likely arose via acquisition of genetic material encoding capsular polysaccharide synthesis machinery by GBS from other streptococcal species. [12]

The greater proportions of serotype VI and VIII-associated disease occurring among Asian individuals in the U.S. is consistent with global serotype distribution patterns: North American and European studies have previously reported that serotypes VI and VIII contribute minimally to the overall burden of both invasive GBS disease and colonization, while studies across East and Southeast Asia report higher frequencies. [1,2,13] In Japan, NVTs have been found to comprise approximately 13% of all invasive disease strains in adults, with serotype VI and VIII contributing 9.5% and 3.8%, respectively. [14] Large-scale, multi-centered, and geographically stratified studies addressing the distribution of invasive GBS across Southeast Asia are lacking. Serotype VI has been found to be the predominant strain of invasive disease in adults in Taiwan at 33%. [15] In Thailand, serotype VI has been found to be the second most common serotype isolated from adults with invasive GBS disease (13.8%). [16] While comprising a notably lower proportion of invasive cases than reported in its neighboring country of Thailand, serotypes VI and VIII combined reportedly accounted for 5% of invasive cases in Malaysia. [17] Even outside of Asia, serotypes VI and VIII can contribute significantly to the overall burden of GBS. Serotype VI has been reported in 9.3% of colonization and invasive cases combined in Saudi Arabia, 12.2% of colonization cases in Egypt, and 8.2% of colonization strains in the Brazilian Amazon. [18–20]

The findings on the emergence of serotypes VI and VIII highlight potential gaps in coverage of GBS vaccines currently in development. The GBS6 vaccine, which targets serotypes Ia, Ib, II, III, IV, and V, is in late-stage clinical development to prevent neonatal GBS disease and may also benefit adults. [6] However, serotypes VI and VIII are not included in this formulation. An alternative hexavalent conjugate vaccine (covering Ia, Ib, II, III, V, and VII) similarly excludes these emerging serotypes, though other vaccine candidates targeting conserved surface proteins may provide cross-serotype protection. [21]

Widespread use of pneumococcal conjugate vaccines has led to shifts in serotype distribution. In some settings, this has included an increase in NVT strains in invasive disease. [22] There are markedly fewer known serotypes for GBS compared to *Streptococcus pneumoniae* (10 vs 100+), and it is unclear what impact potential adult GBS vaccines will have on colonization. Nevertheless, the increase in NVT GBS serotypes in our study, in conjunction with the possibility of additional serotype replacement through vaccine use, warrants attention and has implications for future vaccine development.

For both serotypes VI and VIII, recent invasive isolates were interspersed throughout the phylogenies rather than concentrated within a single clade, indicating that the increase in incidence was not attributable to clonal expansion. Similarly, neither patient race nor geographic location demonstrated strong phylogenetic structuring for either serotype. However, sufficient phylogenetic signaling was observed in serotype VIII to indicate a clade comprised of isolates originating mostly from the Maryland ABCs site (91.7% 22/24). Of the 24 isolates within the observed clade, 29.1% (7/24) were recovered in 2022–2023, consistent with potential ongoing transmission and recent expansion of this Maryland-associated cluster through 2023. Continued large-scale epidemiologic and genomic surveillance will be helpful to monitor this emergence, particularly if GBS vaccines are introduced.

This study has limitations. First, this study only included data from the seven ABCs sites that provided GBS isolates for people of all ages; two of these sites (California and Colorado) provided data for only the latter portion of the study period. It is possible that demographic distributions and trends might differ among non-participating states, as well as among sites included later in this study. However, a sensitivity analysis of disease trends limited to cases from sites common to all study years found no significant change in results. Second, this study only included isolates from invasive GBS disease cases and, therefore, cannot address serotype distribution or trends in non-invasive infections.

Although serotypes VI and VIII remain rare causes of invasive GBS disease among adults, their increasing incidence highlights the need for careful consideration of vaccine formulations to ensure broad protection across diverse populations. Our findings also highlight the value of large-scale epidemiologic and genomic surveillance. While several maternal GBS vaccine candidates are currently in clinical trials, further research may be needed to evaluate the potential of GBS vaccines to reduce disease burden among adults.

## Supporting information

S1 Text Supplemental methods. (PDF)

S1 Fig Maximum likelihood tree of recombination-masked whole-genome alignment of GBS serotype VI isolates from adult invasive disease cases. Rings (inside to outside) represent year of isolate collection, state, and race. Abbreviations: AI/AN, American Indian/Alaska Native; CA, California; CO, Colorado; GA, Georgia; MD, Maryland; MN, Minnesota; NM, New Mexico; OR, Oregon.
(PDF)

S2 Fig Maximum likelihood tree of recombination-masked whole-genome alignment of GBS serotype VIII isolates from adult invasive disease cases. Rings (inside to outside) represent year of isolate collection, state, and race. Abbreviations: AI/AN, American Indian/Alaska Native; CA, California; CO, Colorado; GA, Georgia; MD, Maryland; MN, Minnesota; NM, New Mexico; OR, Oregon.
(PDF)
