# Supplementary material for: Epidemiologic characteristics of invasive group B streptococcal infections caused by rare serotypes among adults in the United States, 2007–2023
Source: PLOS Glob Public Health. 2026 Apr 21;6(4):e0006205. doi: 10.1371/journal.pgph.0006205 (PMC13098976; doi:10.1371/journal.pgph.0006205)
Supplement: S1 Text — (PDF) [file pgph.0006205.s001.pdf]

SUPPLEMENTAL METHODS

Group B *Streptococcus* chromosomal deoxyribonucleic acid preparation, library construction, and WGS generation were performed as previously described [1]. The bioinformatics pipeline responsible for assignments of serotypes and other parameters has been described [2]. Paired-end Illumina reads were pre-processed with Cutadapt v1.6 by calling the '-q 20' option to remove low quality bases from the end of the read, trimming the Illumina TruSeq universal and index adapters with the '-b' argument, and filtering out reads less than 50 bases with the '--minimumlength' option [3]. De novo assembly was carried out with Velvet v1.2.10 as optimized by the VelvetOptimiser v2.2.5 wrapper [4]. Scaffolding was turned off and the Perl script VelvetK (http://bioinformatics.net.au/software.velvetk.shtml) was used to estimate the best kmer length given a 2.2M size genome. Only assemblies that had a contig number <= 200 and N50 >= 30000 were included in the phylogenetic analysis. Further restricting the dataset to only include cases from individuals >= 18 years of age and collected before 2024 yielded 210 GBS serotype VI and 122 GBS serotype VIII genomes available for phylogenetic reconstruction. For each serotype, reads were mapped against the GBS 2603 V/R reference strain using Snippy v4.6.0 and a fulllength alignment was produced using the 'snippy-clean_full_aln' script [5]. Recombinant sequences were removed using the Gubbins v3.4 program with the maximum number of iterations set to 30 ('-i 30') though runs for both serotypes converged before the 10th iteration [6]. A recombination masked alignment was then generated using the 'mask_gubbins_aln.py' script and the GBS 2603 V/R reference sequence was removed. Phylogenetic reconstruction was performed using the IQTree2 program v2.2.6 employing ultrafast bootstrap approximation with the number of replicates set to 1000 ('-B 1000') [7,8]. Substitution model selection was optimized using jModelTest by setting the model argument '-m' to 'TEST'. For both serotypes VI

and VIII, the best substitution model was found to be 'K3Pu+F+I'. For serotype VI, an outlier clade comprising 6 isolates was removed to improve tree resolution. Each phylogenetic tree was annotated by case year of collection, U.S. state of residence, and designated race using patient information attained through ABC surveillance. Tree annotation was performed using the iTOL tool [9].

SUPPLEMENTAL REFERENCES

1. B. J. Metcalf et al., Using whole genome sequencing to identify resistance determinants and predict antimicrobial resistance phenotypes for year 2015 invasive pneumococcal disease isolates recovered in the United States. Clin Microbiol Infect 22, 1002.e1001-1002.e1008 (2016).
2. B. J. Metcalf et al., Short-read whole genome sequencing for determination of antimicrobial resistance mechanisms and capsular serotypes of current invasive Streptococcus agalactiae recovered in the USA. Clin Microbiol Infect 23, 574.e577-574.e514 (2017).
3. M. Martin, Cutadapt removes adapter sequences from high-throughput sequencing reads. EMBnet. journal 17, 10-12 (2011).
4. D. R. Zerbino, E. Birney, Velvet: algorithms for de novo short read assembly using de Bruijn graphs. Genome research 18, 821-829 (2008).
5. T. Seemann, snippy: fast bacterial variant calling from NGS reads. (2015). https://github.com/tseemann/snippy
6. N. J. Croucher et al., Rapid phylogenetic analysis of large samples of recombinant bacterial whole genome sequences using Gubbins. Nucleic Acids Res 43, e15 (2015).
7. B. Q. Minh et al., IQ-TREE 2: New Models and Efficient Methods for Phylogenetic Inference in the Genomic Era. Mol Biol Evol 37, 1530-1534 (2020).
8. D. T. Hoang, O. Chernomor, A. von Haeseler, B. Q. Minh, L. S. Vinh, UFBoot2: Improving the Ultrafast Bootstrap Approximation. Mol Biol Evol 35, 518-522 (2018).

9. I. Letunic, P. Bork, Interactive Tree of Life (iTOL): an online tool for phylogenetic tree display and annotation. Bioinformatics 23, 127-128 (2007).
