# Supplementary figures and images for: Epidemiologic characteristics of invasive group B streptococcal infections caused by rare serotypes among adults in the United States, 2007–2023
Source: PLOS Glob Public Health. 2026 Apr 21;6(4):e0006205. doi: 10.1371/journal.pgph.0006205 (PMC13098976; doi:10.1371/journal.pgph.0006205)

Tree scale: 0.00001000010000100001

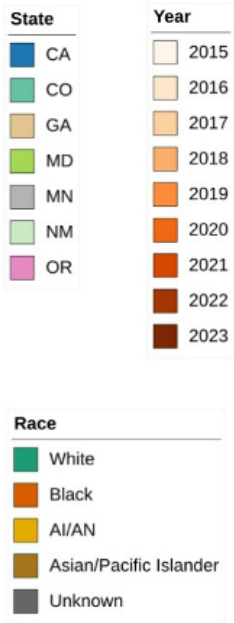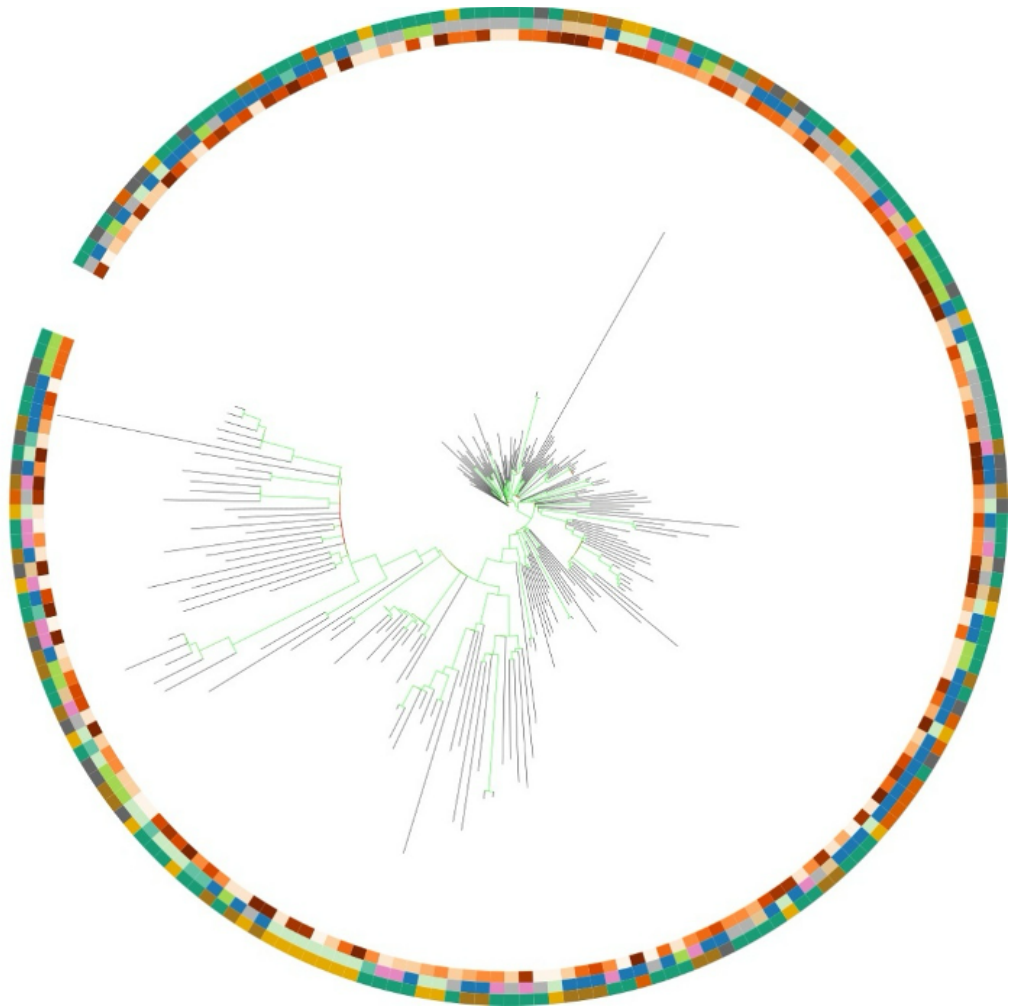

Supplement: S1 Fig — Rings (inside to outside) represent year of isolate collection, state, and race. Abbreviations: AI/AN, American Indian/Alaska Native; CA, California; CO, Colorado; GA, Georgia; MD, Maryland; MN, Minnesota; NM, New Mexico; OR, Oregon. (PDF) [file pgph.0006205.s002.pdf]

Tree scale: 0.00010000100001000009

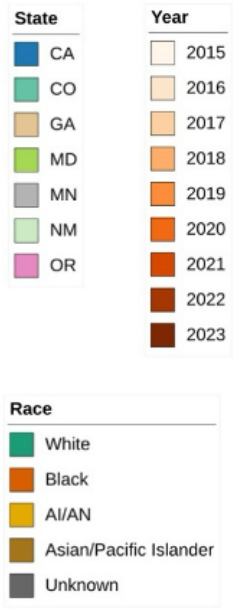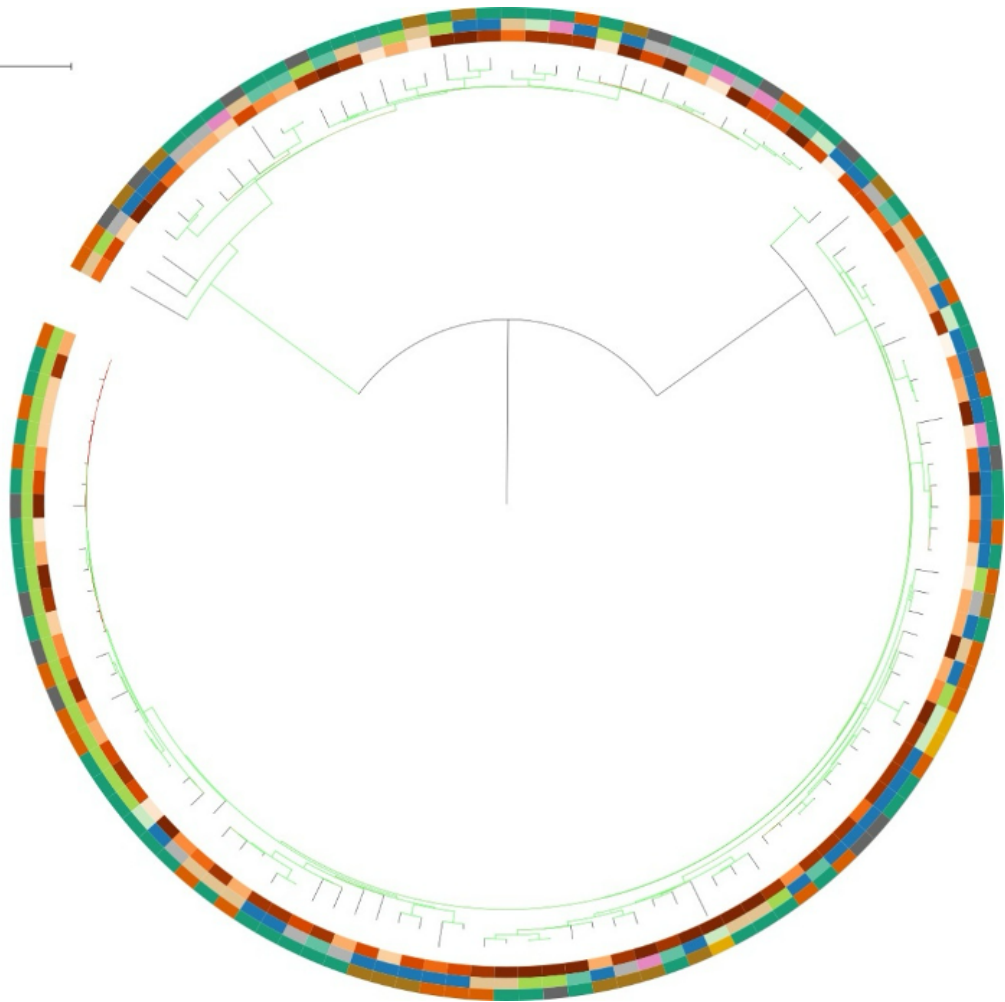

Supplement: S2 Fig — Rings (inside to outside) represent year of isolate collection, state, and race. Abbreviations: AI/AN, American Indian/Alaska Native; CA, California; CO, Colorado; GA, Georgia; MD, Maryland; MN, Minnesota; NM, New Mexico; OR, Oregon. (PDF) [file pgph.0006205.s003.pdf]
